# 

**Published:** 1842-03

**Authors:** 


Volume II. MARCH, 1842. No. 3.

THE

### AMERICAN JOURNAL AND LIBRARY OF Dental Science.

PUBLISHED QUARTERLY.

PUBLISHED UNDER THE AUSPICES OF THE

**American Society of Dental Surgeons.**

EDITED BY

CHAPIN A. HARRIS, M. D., BALTIMORE,

AND

SOLYMAN BROWN, M. D., NEW YORK.

NEW YORK:—GEORGE ADLARD,

BALTIMORE:—ARMSTRONG & BERRY,

LONDON:—SAMUEL HIGHLEY.

WOODS AND CRANE, PRS., BALT.

$5 00 per annum, payable in advance.

This number contains 10 sheets.

